# Neoadjuvant sirolimus for a large hepatic perivascular epithelioid cell tumor (PEComa)

**DOI:** 10.1186/1477-7819-12-46

**Published:** 2014-02-27

**Authors:** Francesca Bergamo, Marco Maruzzo, Umberto Basso, Maria Cristina Montesco, Vittorina Zagonel, Enrico Gringeri, Umberto Cillo

**Affiliations:** 1 Medical Oncology 1 Unit, ISTITUTO ONCOLOGICO VENETO IOV– IRCCS, Via Gattamelata 64, 35128 Padova, Italy; 2 Sarcoma and Melanoma Pathology Unit, ISTITUTO ONCOLOGICO VENETO IOV–IRCCS, Padova, Italy; 3 Hepatobiliary Surgery and Liver Transplantation Unit, University of Padova, Padova, Italy

**Keywords:** Perivascular epithelioid cell, PEComa, neoadjuvant, soft tissue sarcoma, sirolimus

## Abstract

Perivascular epithelioid cell tumors (PEComas) are rare soft-tissue tumors with an extremely heterogeneous clinical behavior. They may arise in different organs and may behave indolently or sometimes metastasize with different grades of biological aggressiveness. We report the case of a young woman with a primary inoperable PEComa of the liver with malignant histological features. Since the mTOR pathway is often altered in PEComas and responses have been reported with mTOR-inhibitors such as sirolimus or temsirolimus, we decided to start a neoadjuvant treatment with sirolimus. The patient tolerated the treatment fairly well and after 8 months a favorable tumor shrinkage was obtained. The patient then stopped sirolimus and 2 weeks later underwent partial liver resection, with complete clinical recovery and normal liver function. The histological report confirmed a malignant PEComa with vascular invasion and negative margins. Then 6 additional months of post-operative sirolimus treatment were administered, followed by regular radiological follow-up. For patients with a large and histologically aggressive PEComa, we think that neoadjuvant treatment with mTOR-inhibitor sirolimus may be considered to facilitate surgery and allow early control of a potentially metastatic disease. For selected high-risk patients, the option of adjuvant treatment may be discussed.

## Background

Sarcomas are a heterogeneous, rare and complex group of mesenchymal tumors, which can occur at any age and in any part of the body. Rarity of the disease, heterogeneity of the site, grade and histology have been the major determinants for the controversial results obtained in clinical studies conducted so far in the management of localized sarcomas [1].

The term perivascular epithelioid cell tumor (PEComa) was first introduced by Zamboni *et al*. [2] to describe a family of soft-tissue tumors characterized by melanocytic and smooth muscle differentiation that may arise in different organs and are considered ubiquitous tumors. The distinctive feature of PEComa is the perivascular epithelioid cell, a mesenchymal cell type frequently seen adjacent to blood vessels*.* The so-called PEComa family of tumors encompasses additional clinical entities such as angiomyolipoma, clear-cell sugar tumors of the lung, lymphangioleiomyomatosis and unusual clear-cell tumors of various organs [3]. Their biological behavior is extremely heterogeneous, ranging from indolent and benign forms to aggressive tumors with malignant transformation and metastatic potential [4].

Due to the rarity and different sites of presentation, the management of these tumors is still a matter of debate in terms of the timing of surgery and the need formultimodal treatments. Here we report the case of a young woman with a primitive PEComa of the liver who underwent radical resection after neoadjuvant treatment with sirolimus.

## Case presentation

**Figure 1:**
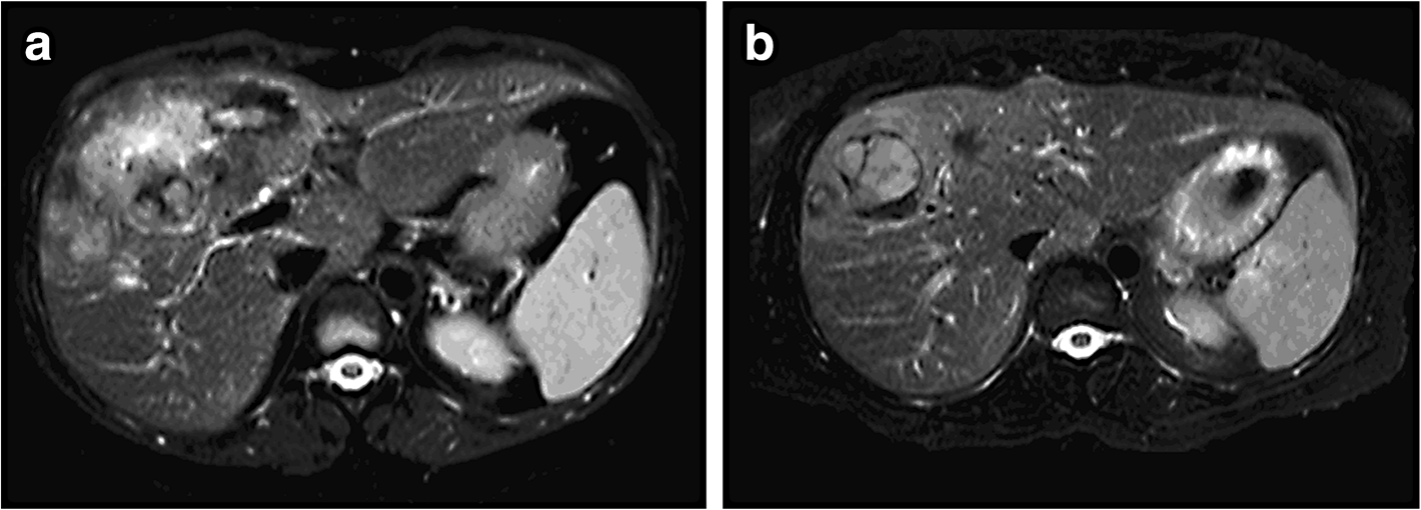
**Liver MRI scans. (a)** At first diagnosis. **(b) **After 8 months of sirolimus, showing a very good radiological response.

**Figure 2:**
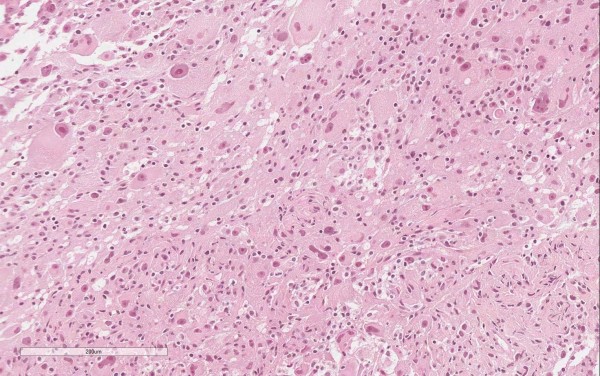
Tumor histology at first diagnosis.

A 31-year-old woman was first referred to our institution in January 2012 because vomiting and gastric reflux had prompted a liver echography and a large hepatic mass had been found. The patient was on an antidepressant drug (ziprasidone) plus lansoprazole. She underwent magnetic resonance imaging (MRI), which showed a voluminous, richly vascularized mass occupying the right lobe of the liver (Figure 1a). The biopsy showed sheets of large epithelioid cells with abundant eosinophilic cytoplasm and pleomorphic nuclei with prominent nucleoli. Scattered multinuclear giant cells were present. Mitotic activity was 4/50 high power fields (HPF) and tumor necrosis was not observed (Figure 2)*.* Immunohistochemically, the tumor cells were strongly positive for MelanA and microphthalmia transcription factor (MIFT), and focally positive for HMB-45, desmin and smooth muscle actin. Lymphovascular invasion was found in the specimen. A diagnosis of epithelioid angiomyolipoma with high-grade cellular atypia (epithelioid PEComa with malignant potential) was therefore made, according to the criteria proposed by Folpe and Kwiatkowski [4].

A total body computed tomography scan excluded the presence of extra-hepatic disease and hematology, renal and liver function tests were normal. Our gastrointestinal Multidisciplinary Team discussed surgical options but in consideration of the volume of the disease, very close to hepatic veins, we decided to postpone surgery and consider neoadjuvant treatment. PEComas are usually considered chemoresistant tumors, but published reports of responses obtained with the mTOR-inhibitors sirolimus and temsirolimus [5-9] provided the rationale for the use of an agent of this class.

Two months later the patient started therapy with oral sirolimus 2 mg per day continuatively, as compassionate use authorized by the local Ethics Committee (Comitato Etico of Istituto Oncologico Veneto (Padova, Italy)). In the absence of toxicity at day 15, the dose was increased to 3 mg per day. Her sirolimus plasma concentration was regularly checked due to the risk that liver involvement by the tumor and concomitant medications could alter drug clearance. Trough values were in the range from 12.6 to 20.1 μg/l, and therefore within therapeutic range. Over the following weeks the patient experienced gastrointestinal toxicity (diarrhea and gastric reflux, grade 2 according to CTCAE), and so loperamide and analgesics were administered and there were a few short treatment interruptions.

After 3 months, an MRI scan demonstrated a partial response of the mass, with colliquation of its inner part and a reduction of the internal vascularization. Thus, sirolimus was continued at the same dosage for another 5 months, when a new radiological assessment showed further shrinkage of the tumor (Figure 1b).

After a multidisciplinary discussion confirmed resectability of the mass, the patient stopped taking sirolimus and 2 weeks later underwent resection of segments IVb, V and VI. The surgical procedure was carried out free of complications, with full recovery. All surgical margins were negative at the final histological examination, which confirmed malignant PEComa with vascular invasion.

The presence of intravascular tumor cells is a strongly adverse prognostic factor for relapse of breast, colon and other types of tumors, while its role in sarcomas is not universally recognized [10]. However, in consideration of the size and the malignant features of this tumor, as well as fairly good tolerability of the drug, we decided to administer 6 additional months of sirolimus as post-operative treatment, still ongoing at the time of this writing.

## Discussion

The definition of perivascular epithelioid cell neoplasia or PEComa encompasses several types of tumors including angiomyolipoma, clear-cell sugar tumors of the lung, lymphangioleiomyomatosis and unusual clear-cell tumors occurring in the kidney, pancreas, uterus or liver, among others [3]. The majority of PEComas arise in women, with a median age of 38 years [6]. They may arise in different anatomical sites, even if they seem to occur with more frequency in the retroperitoneum, abdominal cavity and other viscera such as the gastrointestinal tract, pelvis and liver [3,4].

PEComas are generally benign tumors, and usually do not recur after surgical resection; however, a subgroup of PEComas exhibits a combination of infiltrative growth, atypical mitotic figures, high mitotic index and marked hypercellularity. These lesions have a malignant behavior, with either local recurrence or metastatic spread, commonly in the lungs. Folpe and Kwiatkowski [4] reported an association between malignant clinical behavior and a tumor size greater than 5 cm, histological infiltrative pattern, more than 1 mitosis/50 HPF and a high nuclear grade.

PEComas may be associated with the tuberous sclerosis complex (TSC), an autosomal dominant neurocutaneous disorder caused by genetic alterations of the *TSC1* (9q34) or *TSC2* (16p13.3) genes [11], and characterized by mental retardation, seizures and multiple tumors in different sites [12]. The role of the TSC genes in the pathogenesis of sporadic PEComa has still to be elucidated, but recent data suggest that small deletions or mutations (inactivating or missense) in genes *TSC1* or *TSC2* may account for loss of *TSC2* expression in those tumors [5,13]. For a series of 15 PEComas, Kenerson *et al*. [14] reported the immunohistochemical evidence of mTORC1 activity, which suggests loss of *TSC1* or *TSC2*. The loss of heterozygosity in the *TSC1* or *TSC2* region has also been reported in seven cases of PEComa by Pan *et al*. [15]. These data support inhibition of mTOR as a rational strategy for treating tumors occurring in TSC or those harboring TSC-related mutations.

Due to its rarity, data on medical treatment for PEComas originate from small case series, most of which employed the mTOR-inhibitor sirolimus. Wagner *et al*. treated three patients with sirolimus, all of whom showed a radiographic responses [5]. Bissler *et al*. [7] described a program of 12 months of treatment followed by 12 months of observation for angiomyolipoma, reporting a 53% volume reduction during active therapy, but also 86% regrowth during the observation period. This experience underlines the role of continuative therapy to maintain tumor shrinkage. A case of facial angiofibroma associated with TSC and treated with sirolimus has been published [8]. One significant response has been reported with temsirolimus, an alternative mTOR inhibitor that requires weekly intravenous injections [9].

Our patient was diagnosed with a large and aggressive variant of a PEComa, which showed significant shrinkage after upfront treatment with sirolimus. This allowed radical removal of the mass without surgical complications or sequelae. The tolerability of this agent was fairly good although there were some short treatment breaks due to gastrointestinal toxicity. The sirolimus plasma concentration was regularly checked to ensure that any impairment of liver metabolism due to tumor involvement or interactions with concomitant medications (ziprasidone and analgesics) did not expose the patient to excessive drug concentrations.

## Conclusion

This case is, to our knowledge, the first report of the use of neoadjuvant sirolimus for malignant PEComa achieving a significant impact on patient management and with a possibly curative potential. This confirms the importance of mTOR pathway inhibition in the treatment of these rare tumors. Tumors deemed inoperable or with borderline resectability may therefore be evaluated early for systemic neoadjuvant treatment with sirolimus to obtain tumor shrinkage and facilitate surgical removal. The option of adjuvant treatment may be considered for selected high-risk patients who can tolerate the mTOR inhibitor.

## Consent

Written informed consent was obtained from the patient for publication of this case report and accompanying images. A copy of the written consent is available for review by the Editor-in-Chief of this journal.

## Abbreviations

HPF: high power fields; MRI: magnetic resonance imaging; PEComa: perivascular epithelioid cell tumor; TSC: tuberous sclerosis complex.

## Competing interests

We declare no competing interests for all authors.

## Authors’ contributions

FB, UB, VZ and UC made substantial contributions to conception, data acquisition and analysis and interpretation of results. FB, MM, MCM were involved in drafting the article. UB and EG revised the manuscript. All authors have read and approved the final manuscript.
